# The Health Resources and Services Administration’s Ryan White HIV/AIDS Program in rural areas of the United States: Geographic distribution, provider characteristics, and clinical outcomes

**DOI:** 10.1371/journal.pone.0230121

**Published:** 2020-03-23

**Authors:** Pamela W. Klein, Tanya Geiger, Nicole S. Chavis, Stacy M. Cohen, Alexa B. Ofori, Kathryn T. Umali, Heather Hauck

**Affiliations:** 1 HIV/AIDS Bureau, Health Resources and Services Administration, Rockville, Maryland, United States of America; 2 Federal Office of Rural Health Policy, Health Resources and Services Administration, Rockville, Maryland, United States of America; Brigham and Women's Hospital, UNITED STATES

## Abstract

**Background:**

People living with HIV (PLWH) residing in rural areas experience substantial barriers to HIV care, which may contribute to poor HIV health outcomes, including retention in HIV care and viral suppression. The Health Resources and Services Administration’s Ryan White HIV/AIDS Program (HRSA RWHAP) is an important source of HIV medical care and support services in rural areas. The purpose of this analysis was to (1) assess the reach of the RWHAP in rural areas of the United States, (2) compare the characteristics and funded services of RWHAP provider organizations in rural and non-rural areas, and (3) compare the characteristics and clinical outcomes of RWHAP clients accessing medical care and support services in rural and non-rural areas.

**Methods and findings:**

Data for this analysis were abstracted from the 2017 RWHAP Services Report (RSR), the primary source of annual, client-level RWHAP data. Organizations funded to deliver RWHAP any service (“RWHAP providers”) were categorized as rural or non-rural according to the HRSA FORHP’s definition of modified Rural-Urban Commuting Area (RUCA) codes. RWHAP clients were categorized based on their patterns of RWHAP service use as “visited only rural providers,” “visited only non-rural providers,” or “visited rural and non-rural providers.” In 2017, among the 2,113 providers funded by the RWHAP, 6.2% (n = 132) were located in HRSA-designated rural areas. Rural providers were funded to deliver a greater number of service categories per site than non-rural providers (44.7% funded for ≥5 services vs. 34.1% funded for ≥5 services, respectively). Providers in rural areas served fewer clients than providers in non-rural areas; 47.3% of RWHAP providers in rural areas served 1–99 clients, while 29.6% of non-rural providers served 1–99 clients. Retention in care and viral suppression outcomes did not differ on the basis of whether a client accessed services from rural or non-rural providers.

**Conclusions:**

RWHAP providers are a crucial component of HIV care delivery in the rural United States despite evidence of significant barriers to engagement in care for rural PLWH, RWHAP clients who visited rural providers were just as likely to be retained in care and reach viral suppression as their counterparts who visited non-rural providers. The RWHAP, especially in partnership with Rural Health Clinics and federally funded Health Centers, has the infrastructure and expertise necessary to address the HIV epidemic in rural America.

## Introduction

Nearly one in five Americans live in rural areas of the United States [1]. People who live in rural areas are more likely to be older, poorer, and sicker compared to people living in non-rural areas [2, 3]. Life expectancy also decreases as rurality increases [4]. The provision of healthcare in rural areas is key to addressing the health disparities between rural and non-rural residents of the United States. However, healthcare in rural areas is limited due to comparatively fewer primary healthcare providers, specialists, and dentists [3, 5, 6]. In addition, rural residents often live farther from healthcare providers and service delivery sites than non-rural residents; therefore, transportation to medical facilities and providers is an important barrier to healthcare access [6, 7].

Of the approximately one million people living with diagnosed HIV (PLWH) in the United States in 2017, more than 54,500 (5.9%) reside in rural areas, the majority of whom live in the South (65.2%) [8]. Similar to the demographic profile of all rural residents, PLWH in rural areas are older and more likely to be White that PLWH in non-rural areas. PLWH living in rural areas experience substantial barriers to HIV care, including transportation and long distances to care, provider discrimination and stigma, concerns about confidentiality, lack of health care coverage, and limited healthcare options [9]. These barriers may contribute to delays in HIV testing among rural PLWH and some evidence suggests that rural PLWH are less likely to be retained in care, adhere to antiretroviral medication, and reach viral suppression than PLWH living in non-rural areas [10–15]. Retention in care and viral suppression are key steps along the HIV care continuum; PLWH who are retained in care are more likely to achieve viral suppression, and viral suppression is associated with improved health outcomes [16–18]. PLWH who are unaware of their HIV status or are aware of their status but are not actively engaged in HIV care are more likely to transmit HIV than PLWH who are aware of their HIV status and virally suppressed [19].

Given that healthcare options for PLWH in rural areas may be limited, the Health Resources and Services Administration’s (HRSA) Ryan White HIV/AIDS Program (RWHAP) may be a key component in addressing the healthcare and support services needs of PLWH in rural communities. The RWHAP provides a comprehensive system of HIV primary medical care, medication, and essential support services to more than half a million low-income PLWH each year [20]. RWHAP funds are primarily distributed to grant recipients based on the geographic distribution of PLWH; because the majority of PLWH live in non-rural areas, the majority of RWHAP funding is distributed to grant recipients and subrecipients in non-rural areas. However, the RWHAP is still an important source of HIV medical care and support services in rural areas, particularly through funding to states (RWHAP Part B grants) and local community based organizations (Part C grants).

RWHAP grant recipients, rather than the HRSA HIV/AIDS Bureau (HAB), identifies the service categories that they will deliver to PLWH based on local epidemiologic trends, consumer engagement, and workforce and infrastructure availability. In rural areas of the United States, injection drug use is a major driver of HIV transmission, and rural counties have been identified as most vulnerable to the rapid spread of HIV and hepatitis C (HCV) [8, 21, 22]. Recent outbreaks of HIV and HCV driven by injection opioid use in rural areas suggest that the current U.S. opioid epidemic has the potential to drastically impact the HIV and healthcare environment in many rural communities [23–27]. Therefore, the presence of RWHAP service providers in rural areas at high risk for HIV and HCV outbreaks is crucial to meeting the needs and improving health outcomes for PLWH in rural areas.

The purpose of this analysis was to (1) assess the reach of the RWHAP in rural areas of the United States, (2) compare the characteristics and funded services of RWHAP provider organizations in rural and non-rural areas, and (3) compare the characteristics and clinical outcomes of RWHAP clients accessing medical care and support services in rural and non-rural areas.

## Methods

### RWHAP overview

The RWHAP has five statutorily defined Parts that provide funding for medical and support services, technical assistance, clinical training, and the development of innovative models of care to meet the needs of different communities and populations affected by HIV. The HRSA RWHAP Part A program provides funding to Eligible Metropolitan Areas and Transitional Grant Areas that are most severely affected by the HIV epidemic. The HRSA RWHAP Part B program provides funding to all 50 states, the District of Columbia, Puerto Rico, the U.S. Virgin Islands, and six U. S. Pacific Jurisdictions. The HRSA RWHAP Part B program also awards and administers funding for the AIDS Drug Assistance Program (ADAP) to fund medication and insurance assistance. The HRSA RWHAP Part C program provides funding to local community-based organizations, community health centers, health departments, academic medical centers, and hospitals in the United States, while the Part D program provides funding to support services for low-income women, infants, children, and youth living with HIV and their affected family members.

The Part F program, the fifth statutorily defined Part of the RWHAP, includes the AIDS Education & Training Centers, Special Projects of National Significance, and dental programs.

### Data source

Data for this analysis were abstracted from the 2017 RWHAP Services Report (RSR). The RSR data set is HRSA HAB’s primary source of annual, client-level RWHAP data used to assess the numbers and demographics of clients receiving services, as well as their HIV-related outcomes. Each year, RWHAP Parts A-D grant recipients and subrecipients receive funds to provide core medical or support services and are required to submit data to HRSA; RWHAP Part F data are collected through other mechanisms and are not included within this analysis. De-identified client-level RSR data are submitted by more than 2,000 grant recipients and subrecipients in the United States including the 50 states, the District of Columbia, and three territories (Guam, Puerto Rico, and the U.S. Virgin Islands) [20].

The RWHAP defines “provider” as an organization that is funded to deliver services under specific, statutorily-defined service categories. Therefore, a provider is not a specific individual who delivers services, but, rather, the broader organization funded by the RWHAP to deliver services. This analysis includes all RWHAP provider organizations, regardless of the service categories for which they are funded (i.e., this analysis includes organizations funded to deliver core medical services and support services).

This analysis used two sections of the RSR: the provider report, and the client-level data report for all funded providers and clients served by the RWHAP Parts A, B, C, and D during the 2017 calendar year. The provider report includes basic information about RWHAP providers and the services delivered by the provider under RWHAP contracts. The client-level data report includes information on client demographics, service utilization, and HIV-related clinical outcomes. Data collection through RSR and other RWHAP data sources is a routine program activity and the data are used for program monitoring, improvement, evaluation, and policy purposes only. Therefore it is not human subject research and does not require IRB review and approval.

### Defining rural provider organizations and clients

HRSA’s Federal Office of Rural Health Policy (FORHP) classifies all non-Metro counties, as defined by the Office of Management and Budget, as rural [28]. In addition, HRSA FORHP uses Rural-Urban Commuting Area (RUCA) codes to identify other rural areas; any census tract within metropolitan counties having RUCA codes 4–10 and 132 large area census tracts having RUCA codes 2 or 3 are defined as “rural” [28]. The subset of “rural” areas that comprise 132 large census tracts with RUCA codes 2 or 3 are further defined as “metro-rural.” To identify areas with a combination of low population size and high geographic remoteness, we used the U.S. Department of Agriculture (USDA) frontier and remote (FAR) area codes, which are defined in relation to the time it takes to travel by car to the edges of nearby urban areas [29]. While FAR areas do not correlate directly with HRSA-defined rural areas, they can be considered a subset of HRSA-defined rural areas.

The RSR provider report includes the main address of the provider organization and the address of all service locations. However, due to statutory requirements, the RWHAP does not collect any personally identifiable information about a client, including a client’s residential address. Therefore, this analysis defined “rural” location based on the provider organization’s zip code of their main organizational address.

This rural definition does not account for provider organizations that may have a service location in other zip codes, some of which may be rural. Therefore, this rural definition likely underestimates the reach of the RWHAP in rural areas. To assess geographic location using zip code information, this analysis used a HRSA FORHP-created crosswalk of zip codes that identifies the set of non-metropolitan counties and rural census tracts that comprise rural areas as defined by HRSA.

### Provider characteristics and RWHAP-funded service categories

Characteristics and funded service categories were compared between HRSA-designated rural providers and non-rural providers. Provider characteristics were self-reported by the provider organizations and included provider type (e.g., hospital or university-based clinic, publicly funded community health center, health department), ownership type (e.g., public, private), faith-based organization (yes or no), number of full-time staff, and status of Public Health Service Act Section 330 funding, in addition to RWHAP funding. Section 330 of the Public Health Service Act supports the development and operation of community health centers that provide preventive and primary healthcare services, supplemental health and support services, and environmental health services to medically underserved areas and populations. Many Public Health Service Act Section 330 organizations are Health Centers funded by the HRSA Bureau of Primary Health Care; however, Health Center “Look-Alikes” and Rural Health Clinics do not receive Section 330 funding. Based on data submitted to the RSR, we calculated the number of clients served by the provider (categorized for analysis as 1–99, 100–199, 200–299, 300–399, 400–499, and ≥500 clients).

RWHAP providers can be funded to provide any of the 13 core medical services (e.g., outpatient ambulatory health services, medical case management, oral health care, substance abuse, outpatient care) or 17 support services (e.g., medical transportation, residential substance abuse services, short-term housing) as described in the RWHAP statute [30]. At least 75% of all program funds must be spent on any of the 13 core medical services, while up to the remaining 25% of program funds can be used for any of the 17 support service categories; RWHAP recipients can apply for a waiver to this requirement. Core medical services are consistent with clinical and professional standards, including the Health and Human Services’ Clinical Guidelines for the Treatment of HIV. Support services are intended to support and improve the medical outcomes of PLWH. Services delivered by RWHAP recipients and subrecipients are grounded in evidence-based interventions, evidence-informed interventions, or emerging strategies. For this analysis, we examined the number of service categories for which providers were funded, whether they were funded for core medical services, support services, or both core medical and support services, and the specific service categories for which they were funded.

### Client characteristics and clinical outcomes

We compared the demographic characteristics of RWHAP clients who accessed RWHAP services in rural areas, non-rural areas, or both rural and non-rural areas. Client characteristics included in the analysis were age, race/ethnicity (e.g., Black/African American, Hispanic/Latino, White), gender (i.e., male, female, transgender), household income as a percentage of the federal poverty level (FPL), health care coverage (e.g., private employer, private individual, Medicare, Medicare, no coverage), and housing status (i.e., stable, temporary, unstable). Transmission risk categories were classified based on a gender-stratified, hierarchical categorization adapted from the Centers for Disease Control and Prevention’s (CDC’s) National HIV Surveillance System definitions for transmission categories [31]. Based on service use indicated in the RSR, we also assessed the number of RWHAP providers that clients visited.

The HIV clinical outcomes assessed in this analysis were retention in care and viral suppression. Retention in HIV medical care was defined as PLWH who had at least 2 outpatient ambulatory health service (OAHS) visit dates that were at least 90 days apart in 2017, with the first visit occurring before September 1. Viral suppression was defined as the most recent reported HIV RNA test result of <200 copies/mL.

### Statistical analysis

For provider-level analyses, we assessed the number and percent of RWHAP who met the HRSA definition of rural, overall and stratified by HHS region [32]. To demonstrate the reach of the RWHAP, we conducted a sub-analysis identifying metro-rural providers and providers in frontier and remote areas. This descriptive analysis of the RWHAP’s reach in rural areas was replicated specifically among RWHAP providers funded for outpatient ambulatory health services (i.e. HIV medical care). Additionally, we descriptively compared the characteristics and funded service categories for rural and non-rural providers.

For the client-level analysis, we classified clients as “visited only rural providers,” “visited only non-rural providers,” or “visited rural and non-rural providers” based on their patterns of service use within the RWHAP. We descriptively compared the demographic characteristics of these client categories, as well as retention in HIV care and viral suppression.

## Results

### Distribution of RWHAP providers in rural areas

In 2017, among the 2,113 providers funded for any service category by the RWHAP, 6.2% (n = 132) were located in HRSA-designated rural areas (Table 1). Only 0.1% (n = 2) of RWHAP providers were classified as “metro-rural” and 1.6% (n = 34) were in frontier and remote areas. Among 922 RWHAP providers funded to deliver OAHS, 7.6% (n = 70) were in rural areas, with 0.2% (n = 2) in metro-rural areas and 2.0% (n = 18) in frontier and remote areas.

**Table 1:** Number and percent of HRSA RWHAP providers in rural areas, 2017.

	All RWHAP Providers	
	(n = 2,113)	
	N	%
**Main Provider Location**		
HRSA Rural Designated	132	6.2
Metro-Rural (RUCA 2–3)	2	0.1
Frontier and Remote (FAR 1)	34	1.6

**Fig 1:**
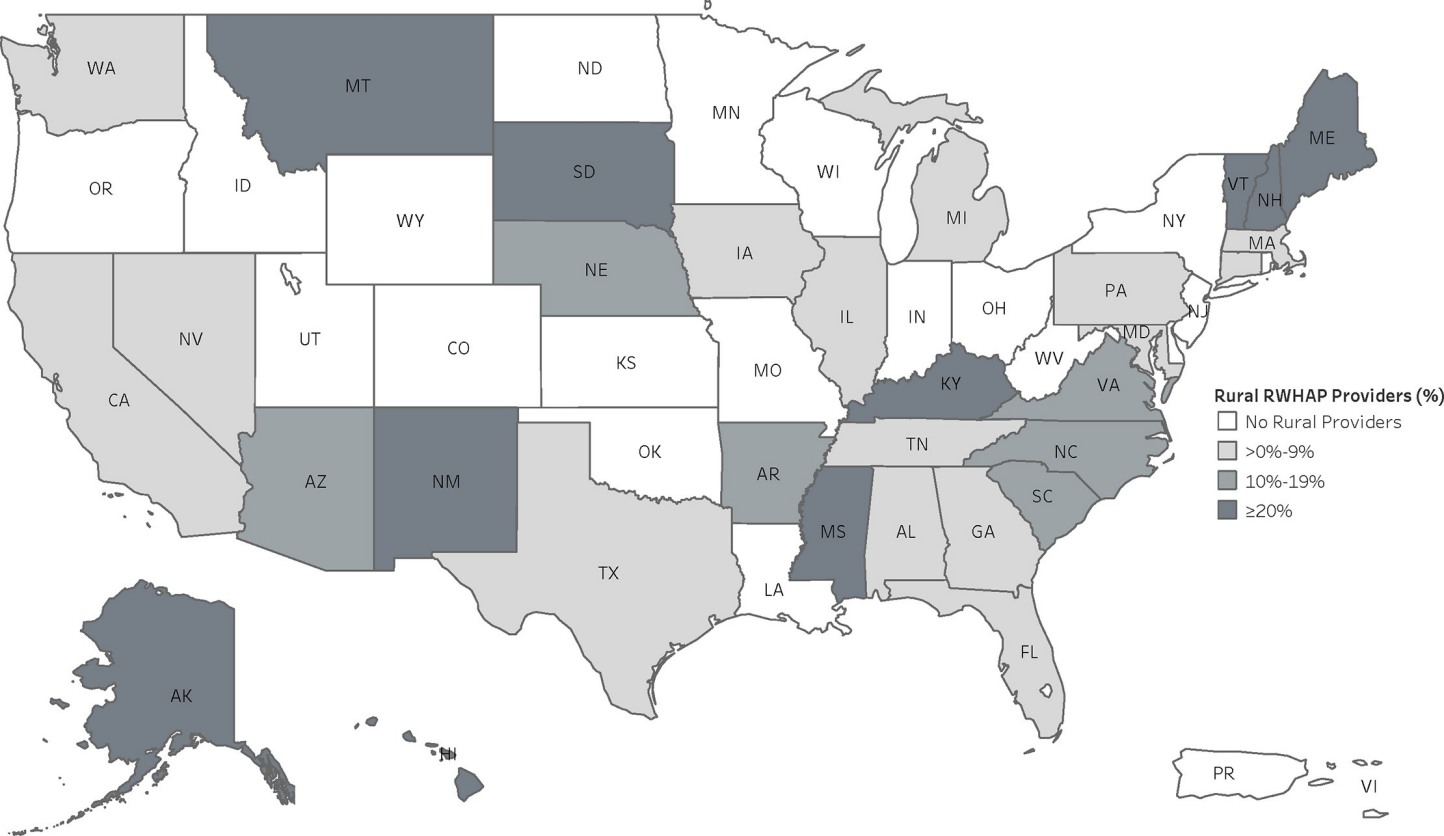
Percent of HRSA RWHAP providers in rural areas, by state, 2017. This figure displays the percentage of RWHAP providers within each jurisdiction that were located in rural-designated areas. The number and percentage of rural providers for all jurisdictions are available in S1 Table.

The distribution of rural RWHAPs providers varied by geography (Fig 1). Over half of states/territories had a provider that was in a rural area (n = 31/54, 57.4%). Among states with rural providers, the proportion of rural providers within the state ranged from a low of 0.8% in Florida (n = 3/361 providers) to a high of 92.0% in New Hampshire (n = 23/25 providers). Five states had more than one-quarter of their providers located in rural areas: Kentucky (33.3%) Montana (70.0%), South Dakota (75.0%), Maine (87.5%), and New Hampshire (92.0%). The number and percentage of rural providers for all states and territories are available in S1 Table.

### Characteristics and funded services of rural and non-rural RWHAP providers

Among RWHAP providers in rural areas, nearly one-third (30.8%) were health department providers, followed by publicly funded community health centers (15.0%), and hospital or university based organizations (10.8%; Table 2). Among RWHAP providers in non-rural areas, 19.8% were hospital or university based organizations, followed by publicly funded community health centers (13.5%) and health department providers (13.1%). A greater proportion of RWHAP providers in rural areas were publicly funded community mental health centers than non-rural providers (3.3% and 0.4%, respectively). However, only 1.7% of rural providers were substance use disorder treatment centers, compared with 2.4% of non-rural providers.

**Table 2:** Characteristics of rural and non-rural HRSA RWHAP providers, 2017.

	Rural Providers		Non-Rural Providers	
	(n = 132)		(n = 1,981)	
	N	%	N	%
**Provider Type**				
Hospital or university-based clinic	13	10.8	317	19.8
Publicly funded community health center	18	15.0	217	13.5
Publicly funded community mental health center	4	3.3	7	0.4
Other community-based service organization	33	27.5	617	38.4
Health department	37	30.8	210	13.1
Substance use disorder treatment center	2	1.7	38	2.4
Solo/group private medical practice	4	3.3	18	1.1
Agency with multiple fee-for-service providers	0	0.0	10	0.6
People living with HIV (PLWH) coalition	0	0.0	2	0.1
VA facility	0	0.0	2	0.1
Other provider type	9	7.5	167	10.4
**Subtotal**	120	100.0	1,605	100.0
**Public Health Service Act Section 330 Funding**				
Yes	116	87.9	1,756	88.6
No	13	9.8	207	10.4
Unknown	3	2.3	18	0.9
**Subtotal**	132	100.0	1,981	100.0
**Ownership Type**				
Public/local	25	20.8	236	14.7
Public/State	19	15.8	156	9.7
Public/Federal	0	0.0	18	1.1
Private, nonprofit	67	55.8	1,083	67.5
Private, for-profit	8	6.7	79	4.9
Unincorporated	0	0.0	2	0.1
Other	1	0.8	30	1.9
**Subtotal**	120	100.0	1,604	100.0
**Faith-Based Organization**				
Yes	1	0.8	68	3.4
No	131	99.2	1,913	96.6
Subtotal	132	100.0	1,981	100.0
**Number of Paid Staff (FTEs)**	3.3		7.7	
**Number of Clients**				
1–99	44	47.3	427	29.6
100–199	23	24.7	289	20.1
200–299	11	11.8	157	10.9
300–399	8	8.6	108	7.5
400–499	2	2.1	69	4.8
500+	5	5.4	390	27.1
**Subtotal**	93	100.0	1,440	100.0

Nearly 90% of RWHAP providers in both rural and non-rural areas received Public Health Service Act Section 330 funding (87.9% and 88.6%, respectively). Over one-third of RWHAP provider organizations in rural areas were publicly owned (36.7%), compared to only 25.5% of provider organizations in non-rural areas. Over half of RWHAP provider organizations in rural areas were owned by a private, non-profit organization (55.8%), compared to 67.5% of provider organizations in rural areas.

RWHAP providers in rural areas had an average of 3.3 paid full-time employees (FTEs) at their organizations, compared with 7.7 FTEs in non-rural providers. Rural providers served a smaller number of RWHAP-eligible clients compared with non-rural providers; 47.3% of RHWAP providers in rural areas served 1–99 clients, while 29.6% of non-rural providers served 1–99 clients.

Overall, RWHAP providers located in rural areas were funded to deliver a greater number of service categories per site than non-rural providers (44.7% funded for ≥5 services vs. 34.1% funded for ≥5 services, respectively; Table 3). Whereas 11.0% of non-rural providers were funded only for support services, 2.3% of rural providers were funded for support services only. Compared to non-rural providers, a greater proportion of rural providers were funded for oral health care (44.7% vs. 27.8%); medical case management (57.6% vs. 47.3%); outpatient ambulatory health services (53.0% vs. 43.0%); early intervention services (24.2% vs. 16.3%); non-medical case management (38.6% vs. 30.7%); emergency financial assistance (25.8% vs. 18.4%); and food bank /home delivered meals (23.5% vs. 15.1%).

**Table 3:** RWHAP funded services by rural and non-rural RWHAP Providers, 2017.

	Rural Providers		Non-Rural Providers	
	(n = 132)		(n = 1,981)	
	N	%	N	%
**Number of Funded Services**				
1	43	32.6	621	31.3
2–5	30	22.7	685	34.6
5–10	36	27.3	399	20.1
11+	23	17.4	276	13.9
**Type of Funded Services**				
Core Only	51	38.6	638	32.2
Core & Support	64	48.5	962	48.6
Support Only	3	2.3	218	11.0
**Core Medical Services Funded**				
AIDS Pharmaceutical Assistance	17	12.9	230	11.6
Early Intervention Services (EIS)	32	24.2	322	16.3
Health Insurance Premium and Cost Sharing Assistance	30	22.7	351	17.7
Home and Community-Based Health Services	4	3.0	69	3.5
Home Health Care	3	2.3	21	1.1
Hospice	0	0.0	7	0.4
Medical Case Management	76	57.6	938	47.3
Medical Nutrition Therapy	30	22.7	317	16.0
Mental Health Services	48	36.4	696	35.1
Oral Health Care	59	44.7	551	27.8
Outpatient/Ambulatory Health Services	70	53.0	852	43.0
Substance Abuse Outpatient Care	14	10.6	273	13.8
**Support Services Funded**				
Child Care Services	0	0.0	18	0.9
Emergency Financial Assistance	34	25.8	364	18.4
Food Bank/Home Delivered Meals	31	23.5	299	15.1
Health Education/Risk Reduction	15	11.4	277	14.0
Housing	13	9.8	220	11.1
Legal Services	0	0.0	0	0.0
Linguistic Services	10	7.6	130	6.6
Medical Transportation	52	39.4	683	34.5
Non-Medical Case Management	51	38.6	608	30.7
Other Professional Services	3	2.3	89	4.5
Outreach Services	23	17.4	290	14.6
Permanency Planning	0	0.0	0	0.0
Psychosocial Support Services	17	12.9	341	17.2
Referral for Health Care and Support Services	8	6.1	194	9.8
Rehabilitation Services	0	0.0	2	0.1
Respite Care	0	0.0	3	0.2
Substance Abuse Services (residential)	0	0.0	57	2.9

### RWHAP clients visiting rural and non-rural providers

Of the 534,802 RWHAP clients who visited a provider in 2017, 12,414 (2.3%) visited only rural providers, 517,877 (96.8%) visited only non-rural providers, and 4,511 (0.8%) visited both rural and non-rural providers (Table 4). Clients who visited only rural providers were slightly older (61.1% were 45 years of age or older) than clients who visited non-rural or visited both types of providers (57.8% and 57.9% were 45 years of age or older, respectively). Approximately 40% of clients who visited only rural providers were White, compared with 25.8% of clients who visited only non-rural providers and 34.7% of clients who visited both rural and non-rural providers. The gender and transmission risk category distributions were similar for clients who visited rural, non-rural, and a mixture of providers.

**Table 4:** Characteristics of RWHAP clients visiting only rural providers, only non-rural providers, and both rural and non-rural providers, 2017.

	Visited Only Rural Providers		Visited Only Non-Rural Providers		Visited Rural and Non-Rural Providers	
	(n = 12,414)		(n = 517,877)		(n = 4,511)	
	N	%	N	%	N	%
**Age group (yr)**						
<13	23	0.2	4,947	1.0	3	0.1
13–24	501	4.1	22,876	4.4	168	3.7
25–34	1,969	15.9	91,070	17.6	793	17.5
35–44	2,327	18.7	99,714	19.2	932	20.7
45–54	3,768	30.3	147,218	28.4	1,372	30.4
55–64	2,895	23.3	116,827	22.6	966	21.4
≥65	931	7.5	35,225	6.8	277	6.1
**Subtotal**	12,414	100.0	517,877	100.0	4,511	100.0
**Race/ethnicity**						
American Indian/Alaska Native	223	1.8	2,648	0.5	40	0.9
Asian	68	0.5	7,293	1.4	22	0.5
Black/African American	5,305	42.7	242,801	46.9	2,062	45.7
Hispanic/Latino^a^	1,603	12.9	120,577	23.3	776	17.2
Native Hawaiian/Pacific Islander	29	0.2	915	0.2	13	0.3
White	5,030	40.5	133,874	25.8	1,567	34.7
Multiple races	150	1.2	6,322	1.2	31	0.7
**Subtotal**	12,408	100.0	514,430	100.0	4,511	100.0
**Gender**						
Male	8,898	71.7	368,155	71.1	3,176	70.4
Female	3,406	27.4	140,361	27.1	1,279	28.4
Transgender MTF	106	0.9	8,189	1.6	50	1.1
Transgender FTM	4	0.0	934	0.2	6	0.1
Transgender unknown	3	0.0	183	0.0	0	0.0
**Subtotal**	12,417	100.0	517,822	100.0	4,511	100.0
**Transmission risk category**						
** Male client**						
Male-to-male sexual contact	5,261	63.7	212,406	64.7	2,065	65.9
Injection drug use	470	5.7	20,138	6.1	147	4.7
Male-to-male sexual contact and injection drug use	322	3.9	10,603	3.2	134	4.3
Heterosexual contact^b^	2,086	25.3	78,528	23.9	742	23.7
Perinatal infection	60	0.7	4,351	1.3	21	0.7
Other^c^	58	0.7	2,081	0.6	24	0.8
**Subtotal**	8,257	100.0	328,107	100.0	3,133	100.0
** Female client**						
Injection drug use	251	7.9	10,461	8.5	78	6.3
Heterosexual contact^b^	2,813	88.9	105,357	85.9	1,131	90.7
Perinatal infection	63	2.0	5,332	4.3	30	2.4
Other^c^	38	1.2	1,518	1.2	8	0.6
**Subtotal**	3,165	100.0	122,668	100.0	1,247	100.0
** Transgender client**						
Sexual contact^e^	97	96.0	6,879	91.6	51	98.1
Injection drug use	1	1.0	137	1.8	0	0.0
Sexual contact^e^ and injection drug use	1	1.0	385	5.1	1	1.9
Perinatal infection	1	1.0	67	0.9	0	0.0
Other^c^	1	1.0	40	0.5	0	0.0
**Subtotal**	101	100.0	7,508	100.0	52	100.0
**Federal poverty level**^**f**^						
0–100%	6,419	51.7	300,744	58.1	2,964	65.7
101–138%	1,614	13.0	55,325	10.7	544	12.1
139–250%	2,268	18.2	78,945	15.3	737	16.4
251–400%	916	7.4	30,805	5.9	216	4.8
>400%	207	1.7	12,278	2.3	19	0.4
**Subtotal**	11,424	100.0	478,097	100.0	4,480	100.0
**Health care coverage**^**f**^						
Private employer	1,119	9.0	47,217	9.6	246	5.5
Private individual	1,259	10.1	37,455	7.6	416	9.2
Medicare	1,486	12.0	52,590	10.6	348	7.7
Medicaid	2,473	19.9	163,127	33.0	839	18.6
Medicare and Medicaid	1,115	9.0	37,721	7.6	467	10.4
Veterans Administration	32	0.3	1,305	0.3	5	0.1
Indian Health Service	18	0.1	202	0.0	9	0.2
Other plan	138	1.1	8,800	1.8	38	0.8
No coverage	2,233	18.0	99,560	20.2	1,215	27.0
Multiple coverages	1,767	14.2	46,068	9.3	920	20.4
**Subtotal**	11,640	100.0	494,045	100.0	4,503	100.0
**Housing status**						
Stable	10,653	85.8	421,604	81.4	3,779	83.8
Temporary	568	4.6	38,181	7.4	454	10.1
Unstable	280	2.3	25,087	4.8	262	5.8
**Subtotal**	11,501	100.0	484,872	100.0	4,495	100.0
**Number of providers visited**						
1	10,708	86.2	390,160	75.3	0	0.0
2	1,371	11.0	89,093	17.2	3,283	72.8
3	313	2.5	27,318	5.3	880	19.5
4	25	0.2	8,396	1.6	255	5.7
5+	0	0.0	3,008	0.6	93	2.1
**Subtotal**	12,417	100.0	517,975	100.0	4,511	100.0

Abbreviations: MTF, male–to–female; FTM, female–to–male.

^a^ Hispanics/Latinos can be of any race.

^b^ Heterosexual contact with a person known to have, or to be at high risk for, HIV infection.

^c^ Includes hemophilia and blood transfusion.

^d^ Subtotals are reflective of available gender and transmission risk category information. The values may not sum to the subtotals for gender overall.

^e^ Includes any sexual transmission risk category reported by transgender clients.

^f^ Subtotals for each subpopulation are displayed to reflect the denominator used for the percentage calculation of each subpopulation; due to missing data, the values in each column may not sum to the column total.

Nearly two-thirds (65.7%) of RWHAP clients who visited both rural and non-rural providers had a household income at or below 100% FPL, whereas 51.7% of clients who visited only rural providers and 58.1% of clients who visited only non-rural providers were at or below 100% FPL. Overall, the most common health care coverage type among RWHAP clients seeking care from rural, non-rural, and both rural and non-rural providers was Medicaid (19.9%; 33%; and 18.6%, respectively). Clients who visited both rural and non-rural providers were more likely to lack health care coverage (27.0%) than clients who visited only rural or only non-rural providers (18.0% and 20.2%, respectively). Clients who visited providers in non-rural areas had slightly lower levels of stable housing (81.4%) than clients who visited providers in rural areas (85.8%) or clients who visited providers in both rural and non-rural areas (83.8%).

### Retention in care and viral suppression among RWHAP clients

RWHAP clients who visited only rural providers had slightly higher rates of retention in care (82.9%, n = 6,246/7,536) than clients who visited non-rural providers (80.8%, n = 266,937/330,356) or clients who visited both rural and non-rural providers (81.4%, n = 2,993/3,678; Table 5). The proportion of RWHAP clients reaching viral suppression was consistent, regardless of where RWHAP clients accessed RWHAP services. Among clients who visited only rural providers, 85.5% (n = 6,718/7,855) reached viral suppression, compared to 85.9% (n = 296,132/344,726) of clients who visited only non-rural clients and 85.9% (n = 3,261/3,796) of clients who visited both rural and non-rural RWHAP providers.

**Table 5:** Retention in care and viral suppression among RWHAP clients, 2017.

	Total No.	Retained		Total No.	Virally Suppressed	
		No.	%		No.	%
Visited Only Rural Providers	7,536	6,246	82.9	7,855	6,718	85.5
Visited Only Non-Rural Providers	330,356	266,937	80.8	344,726	296,132	85.9
Visited Rural and Non-Rural Providers	3,678	2,993	81.4	3,796	3,261	85.9

Retention in care was based on data for PLWH who had at least 1 outpatient ambulatory health services visit by September 1 of the measurement year, with a second visit at least 90 days after.

Viral suppression was based on data for PLWH who had at least 1 outpatient ambulatory health services visit during the measurement year and whose most recent viral load test result was <200 copies/mL.

## Discussion

Of the more than 2,000 provider organizations funded by the RWHAP in 2017, approximately 6% of them were located in rural areas of the United States. Rural providers were funded to deliver more RWHAP service categories than non-rural providers, especially medical and support services such as oral health care and case management, and 87.9% were dually funded by Section 330 of the Public Health Service Act. This suggests that the RWHAP in rural areas not only acts as a critical component of the HIV healthcare delivery system, but also has the potential to leverage resources and expertise of the federal Health Center Program.

Although 5.9% of diagnosed PLWH live in rural areas of the United States, fewer than 4% of RWHAP clients visited rural providers: 2.3% visited only rural providers and 0.8% visited both rural and non-rural providers [8]. PLWH living in rural areas who do not access services from rural providers may not be engaged in care, or may access services from non-rural providers. The demographic characteristics of RWHAP clients who visited only rural RWHAP providers were similar to the overall demographic profile of Americans living in rural areas of the United States—compared with RWHAP clients who visited only non-rural providers, they were older, less likely to be a member of a racial or ethnic minority, and more likely to be living below the federal poverty level. The identification of these sociodemographic differences may inform initiatives designed for certain key populations, such as initiatives to meet the needs of older PLWH who access care in rural areas.

Although previous studies have shown that rural PLWH experience a multitude of barriers to accessing and remaining engaged in HIV care, only some studies found an association between rurality and HIV clinical outcomes [12–15, 33, 34]. Within the RWHAP, these barriers do not appear to negatively impact the HIV clinical outcomes of RWHAP clients. That is, rates of retention in HIV care and viral suppression among RWHAP clients visiting rural providers were comparable to the 97% of clients who visited only non-rural providers. While this may be indicative of the strength of the RWHAP comprehensive system of care, it may also be due to the role of confounding by demographic characteristics such as age. For example, RWHAP clients who visited only rural providers were older than clients who visited only non-rural providers, and older PLWH are more likely to reach viral suppression than younger PLWH [20, 35].

Although RWHAP clients who accessed care in rural areas experienced comparable retention in care and viral suppression to RWHAP clients who accessed care in non-rural areas, this analysis does not address the barriers that rural PLWH may face before they successfully and routinely engage in the healthcare system. Rural PLWH are more likely to delay HIV testing and receive an HIV diagnosis at later disease stages than their non-rural counterparts [10–13]. PLWH who are unaware of their infection account for 38% of all HIV transmissions; those aware of their infection but not in care account for another 42% of all HIV transmissions [19]. Therefore, while this analysis demonstrated that nearly all PLWH accessing care from rural RWHAP are retained in care and reached viral suppression, lack of access to HIV testing services or linkage to HIV care services for newly diagnosed PLWH in rural areas could contribute to continued HIV transmission within rural areas.

RWHAP clients were classified based on their patterns of rural and non-rural service utilization, which may or may not reflect a client’s residence in a rural or non-rural area. However, service utilization patterns offer important insight into how the RWHAP meets the needs of clients in rural and non-rural areas. PLWH residing in rural areas face decisions about where to access their HIV medical care and support services. Evidence suggests that almost three-quarters of PLWH residing in rural areas access healthcare in non-rural areas [21]. Underpinning the decision process of PLWH regarding where they access their HIV medical care and support services are structural barriers like transportation and long distances to care, provider discrimination and stigma, concerns about confidentiality, and lack of health care coverage [9]. Rural areas of the United States may lack extensive public transit systems, which could create barriers to healthcare for individuals who may not have access to or may not be able to afford a private vehicle. Nearly 40% of RWHAP providers in rural areas were funded for medical transportation services, which specifically address this issue. Additionally, societal barriers such as stigma and risks to confidentiality in smaller communities could lead PLWH residing in rural areas to seek care outside of their immediate rural community [9, 36, 37]. Those who experience perceived or real HIV-related stigma and discrimination who do not have the means, transportation, time, or childcare to travel to non-rural areas may fall out of HIV care.

Provider organizations may have multiple locations where they deliver services, some of which may be located in rural areas. However, in this analysis, they would be classified as rural only if their primary address was located in an HRSA-defined rural area. Although the RSR does collect information on where provider organizations deliver services, provider characteristics and patterns of client service use are only collected based on the main provider address. Additionally, organizations not funded by the RWHAP also deliver medical care and support services to PLWH. Therefore, while not captured in this analysis, the RWHAP and broader HIV system of care likely has a larger presence in rural areas than estimated in this analysis.

With the rise of opioid use and associated HIV and HCV outbreaks, identifying the presence of the RWHAP’s comprehensive system of care is crucial to preparing and responding to the intersecting epidemics of opioids and infectious diseases. Within the RWHAP, substance abuse services, case management, and mental health services are especially key to addressing the opioid crisis among PLWH. RWHAP grant recipients select the service categories that they will fund based on local needs and epidemiologic trends, including outbreak response, local infrastructure, and other health care payors. Over one-third of rural RWHAP providers were funded to deliver mental health services, over 10% were funded to deliver substance abuse services, and the majority were funded to deliver case management services. With its presence in rural areas, the RWHAP is well positioned to respond to the needs of these emerging, opioid-impacted communities.

Developing economically viable service delivery programs in rural communities is difficult due to low population density and a lower HIV prevalence than non-rural areas. The RWHAP invests in the identification of new methods and approaches, as well as the expansion of existing approaches, to ensure that PLWH in rural areas have access to high quality HIV care and treatment, including telehealth technology using the Extension for Community Healthcare outcomes (ECHO) collaborative model [38]. The RWHAP’s AIDS Education and Training Centers (AETCs) coordinate the AETC Telehealth Training Centers Program, support multiple ECHO projects, and coordinate a Rural Health Committee, focused specifically on increasing access to HIV care in rural areas [39–43]. In addition to the efforts of the RWHAP, the special healthcare provider designations provide primary care in rural areas and allow organizations access to enhanced payments under Medicare and Medicaid, such as the Essential Community Providers designation, the Rural Health Clinic designation, and the federally-funded Health Center designation [3]. HRSA’s FORHP also supports the Rural Health Information Hub, which offers a library of resources; many of these resources specifically focus on HIV in rural areas, including the “Rural HIV/AIDS Prevention Toolkit” [44].

RWHAP providers are a crucial component of HIV care delivery in the rural United States. Despite evidence of significant barriers to engagement in care for rural PLWH, RWHAP clients who visited rural providers were just as likely to be retained in care and virally suppressed as their counterparts who visited non-rural providers. The RWHAP, especially in partnership with Rural Health Clinics and federally-funded Health Centers, has the infrastructure and expertise necessary to address the HIV epidemic in rural America.

## Supporting information

S1 Table Percent of HRSA RWHAP providers in rural areas, by state, 2017. (DOCX)
