# Supplementary material for: The Health Resources and Services Administration’s Ryan White HIV/AIDS Program in rural areas of the United States: Geographic distribution, provider characteristics, and clinical outcomes
Source: PLoS One. 2020 Mar 23;15(3):e0230121. doi: 10.1371/journal.pone.0230121 (PMC7089565; doi:10.1371/journal.pone.0230121)
Supplement: S1 Table — (DOCX) [file pone.0230121.s001.docx]

**S1 Table. Percent of HRSA RWHAP providers in rural areas, by state, 2017.**

|  | **Rural Providers** | |  | **Total Providers** |
| --- | --- | --- | --- | --- |
|  | **N** | % |  | **N** |
| **States** |  |  |  |  |
| Alabama | 1 | 4.5 |  | 22 |
| Alaska | 1 | 20.0 |  | 5 |
| Arizona | 3 | 11.5 |  | 26 |
| Arkansas | 1 | 12.5 |  | 8 |
| California | 17 | 7.1 |  | 241 |
| Colorado | 0 | 0.0 |  | 31 |
| Connecticut | 3 | 5.7 |  | 53 |
| Delaware | 0 | 0.0 |  | 6 |
| District of Columbia | 0 | 0.0 |  | 24 |
| Florida | 3 | 0.8 |  | 361 |
| Georgia | 1 | 2.8 |  | 36 |
| Hawaii | 2 | 25.0 |  | 8 |
| Idaho | 0 | 0.0 |  | 6 |
| Illinois | 5 | 5.1 |  | 99 |
| Indiana | 0 | 0.0 |  | 29 |
| Iowa | 2 | 8.0 |  | 25 |
| Kansas | 0 | 0.0 |  | 8 |
| Kentucky | 3 | 33.3 |  | 9 |
| Louisiana | 0 | 0.0 |  | 28 |
| Maine | 7 | 87.5 |  | 8 |
| Maryland | 3 | 5.5 |  | 55 |
| Massachusetts | 1 | 1.6 |  | 61 |
| Michigan | 3 | 8.6 |  | 35 |
| Minnesota | 0 | 0.0 |  | 23 |
| Mississippi | 6 | 25.0 |  | 24 |
| Missouri | 0 | 0.0 |  | 28 |
| Montana | 7 | 70.0 |  | 10 |
| Nebraska | 2 | 15.4 |  | 13 |
| Nevada | 1 | 4.2 |  | 24 |
| New Hampshire | 23 | 92.0 |  | 25 |
| New Jersey | 0 | 0.0 |  | 95 |
| New Mexico | 1 | 25.0 |  | 4 |
| New York | 0 | 0.0 |  | 150 |
| North Carolina | 9 | 13.8 |  | 65 |
| North Dakota | 0 | 0.0 |  | 13 |
| Ohio | 0 | 0.0 |  | 37 |
| Oklahoma | 0 | 0.0 |  | 5 |
| Oregon | 0 | 0.0 |  | 12 |
| Pennsylvania | 3 | 3.2 |  | 93 |
| Rhode Island | 0 | 0.0 |  | 4 |
| South Carolina | 5 | 18.5 |  | 27 |
| South Dakota | 3 | 75.0 |  | 4 |
| Tennessee | 3 | 8.8 |  | 34 |
| Texas | 5 | 6.6 |  | 76 |
| Utah | 0 | 0.0 |  | 5 |
| Vermont | 2 | 25.0 |  | 8 |
| Virginia | 5 | 11.4 |  | 44 |
| Washington | 1 | 5.9 |  | 17 |
| West Virginia | 0 | 0.0 |  | 4 |
| Wisconsin | 0 | 0.0 |  | 12 |
| Wyoming | 0 | 0.0 |  | 1 |
|  |  |  |  |  |
| **Territories** |  |  |  |  |
| Guam | 0 | 0.0 |  | 1 |
| Puerto Rico | 0 | 0.0 |  | 69 |
| U.S. Virgin Islands | 0 | 0.0 |  | 2 |
